# Menstrual Blood Donation for Endometriosis Research: A Cross-Sectional Survey on Women’s Willingness and Potential Barriers

**DOI:** 10.1007/s43032-024-01481-3

**Published:** 2024-02-28

**Authors:** Kheira Bouzid, Mathilde Bourdon, Roxane Bartkowski, Marie Verbanck, Charles Chapron, Louis Marcellin, Frederic Batteux, Pietro Santulli, Ludivine Doridot

**Affiliations:** 1 Université Paris Cité, Institut Cochin, Inserm, CNRS, 75014 Paris, France; 2 Département de Gynécologie, Obstétrique Et Médecine de La Reproduction, AP-HP, Centre Hospitalier Universitaire (CHU) Cochin, 75014 Paris, France; 3 UR 7537 - BioSTM Biostatistique, Traitement et Modélisation des données Biologiques, Faculté de Pharmacie de Paris, Université Paris Cité, F-75270 Paris, France; 4 Service d’Immunologie Biologique, AP-HP, Centre Hospitalier Universitaire (CHU) Cochin, 75014 Paris, France

**Keywords:** Endometriosis, Menstrual blood, Biological research, Online survey

## Abstract

An anonymous online survey in French was used to assess if endometriosis patients would be as ready as unaffected women to donate their menstrual blood for biological research on endometriosis and evaluate potential barriers to such donation. It was distributed in September 2022 by social media and two mailing lists, including a French patient organization. The questionnaire assessed participant age and brief medical history (hormonal contraception, endometriosis diagnosis, type of endometriosis), menstrual experience (menstrual blood abundance, dysmenorrhea), and whether participants would donate menstrual blood. Women who self-declared with an established endometriosis diagnosis versus no endometriosis were compared. Seven hundred seventy-eight women answered the survey. Among women with menstruation (*n* = 568), 78% are willing to donate menstrual blood for research. Importantly, this proportion was higher in women who declared having an established endometriosis diagnosis (83%, *n* = 299) compared to self-declared unaffected women (68%, *n* = 134, *p* < 0.001). The previous use of a menstrual cup and dysmenorrhea were significantly associated with the willingness to donate menstrual blood, while the use of hormonal contraception was significantly associated with an unwillingness to donate. Only the previous use of the menstrual cup had a predictive value for menstrual blood donation. No significant relationship was observed between menstrual blood donation and age, heavy menstrual bleeding and in endometriosis patients, endometriosis subtypes. In conclusion, women affected or not by endometriosis are largely willing to donate their menstrual blood for research on endometriosis, dysmenorrhea is not a barrier for donation, and women who use a menstrual cup are the more likely to donate.

**Supplementary Information:**

The online version contains supplementary material available at 10.1007/s43032-024-01481-3.

## Introduction

Endometriosis is a common gynecologic disease defined by the presence of lesions of functional endometrial tissue outside the uterine cavity. It affects around 10% of women in their reproductive years (from puberty to menopause). Symptoms include pelvic pain (dysmenorrhea and chronic), dyspareunia (pain during intercourse), and reduced fertility [1, 2]. Endometriosis is also associated with an increased risk of miscarriage and pregnancy complications [3, 4]. As the symptoms are not specific, the diagnosis of endometriosis is difficult. As a result, there is a delayed diagnosis, estimated to be 8 years on average [5]. Endometriosis strongly affects the quality of life of patients (19% lower than a perfectly healthy adult) [6]. It is an economic burden, with a cost estimated at»10 k€ per year per patient [6]. There is neither specific nor curative medical treatment, and 15–30% of patients do not respond to the available options (contraceptives) [7]. Surgical lesion excision and assisted reproductive technologies are used for drug-resistant pain and infertility, but the recurrence rate remains high (around 20% at 2.5 years) [8]. Endometriotic lesions are thought to come from retrograde menstruation, a reflux of menstrual blood through the fallopian tubes into the peritoneal cavity during the menses. But as this phenomenon occurs in 90% of menstruating women and only 1 in 10 develops endometriosis, other mechanisms are at play, notably immune dysfunction [9, 10]. Little is known about how endometriosis evolves over time. Longitudinal studies and tools allowing such studies are cruelly lacking.

Menstrual blood is an easily accessible biological fluid, available every month in non-pregnant women of reproductive age, without the need for any invasive procedure (not even a needle). One disadvantage is that it is only collectable during the menstruation, so only a few days every month, and it is completely unavailable in case of amenorrhea (which can be medically induced in the treatment of endometriosis, for example). Among the naturally available biological fluids, it is by far one of the least studied. A PubMed search on July 2023 led to 20 to 109 times fewer results for menstrual blood than other biological fluids (1515 articles found for human menstrual fluid/blood/effluent, compared to 30,588 to 166,366 for peripheral blood, saliva, urine samples, feces/stool, or seminal fluid/sperm). This illustrates that menstrual blood was so far greatly overlooked as a biological fluid. A concern may be that menstrual blood is difficult to obtain because of its intimate nature and the taboo around it, but a study with almost 100 women from the UK showed that 78% of women are ready to donate menstrual blood [11]. There may be regional disparities as a similar study conducted in India with almost 500 female healthcare professionals showed only 54% would be willing to donate menstrual blood [12]. However, the willingness to donate menstrual blood of patients suffering from endometriosis was never evaluated. 

Increased popularity of reusable items of personal feminine hygiene products, such as the menstrual cup, makes menstrual blood very easy to collect. The menstrual blood volume is around 50–100 mL per cycle with 80% of it being lost during the first 3 days of the cycle [13], allowing to easily collect 2–8 mL from a cup worn 4 h on the heaviest day. This collection method greatly improves the possibility to study menstrual cells and secreted factors in the menstrual serum. Menstrual blood contains viable immune cells and endometrial cells (both stromal and epithelial cells) [14–16]. Interestingly, menstrual immune cells are more similar to the uterine microenvironment than the circulating immune cells found in the peripheral blood [14], with less T lymphocytes and more natural killer (NK) cells as it is observed in the uterus.

While menstrual blood is easily accessible and relevant to both gynecological disorders and fertility, there are few studies in this biological fluid in these pathological contexts. Most studies focused on its use as a source of mesenchymal stem cells for regenerative therapies [17]. While a few studies [16, 18–23] already used menstrual blood from endometriosis patients, the number of donating patients was always limited (6 to 13), so their general willingness to donate remains unknown. So our main objectives in this study were to evaluate the willingness of endometriosis patients to donate menstrual blood, as compared to unaffected women, and to identify potential barriers to such donation. Identifying such barriers is important to assess potential bias in future research using this biological fluid (i.e., would collecting menstrual blood for endometriosis research only capture a subpopulation of endometriosis patients?).

Here, we evaluated the willingness to donate menstrual blood in women who self-declared as affected or not by endometriosis in a short online survey. We looked at the characteristics of our population (such as age, menstruation, endometriosis) and assessed how the different variables (hormonal contraception, endometriosis, type of endometriosis, menstrual blood abundance, dysmenorrhea) correlated to one another. We also looked for predictive factor for menstrual blood donation willingness.

## Materials and Methods

### Participants and Recruitment

A LimeSurvey public online survey (written in French) was distributed through an institutional mailing list (at Institut Cochin), a post on social media (LinkedIn, LD’s account), and through a patient organization (EndoFrance) with a short introduction on the topic and the possibility for people to share the survey more broadly. The diffusion through the research institution mailing list (Institut Cochin) reached its around 600 employees (students, technicians, clinicians, researchers, and admin staff). EndoFrance mailing list reached their 1754 members. In all instances, individuals had the possibility to share the survey link to their own networks. This way, it was notably republished by 23 other LinkedIn accounts. The link to the survey was active from September 2022 to February 2023. No incentive was provided for questionnaire completion. The short introduction indicated that (1) the questionnaire was about menstruation and endometriosis research, (2) it was not necessary to be affected by this disease to answer, and (3) the survey was addressed to women with menstruation. The survey was anonymous and conducted according to French law and European General Data Protection Regulation. A total of 828 questionnaires were accessed, including 778 with at least one completed question. A timestamp was available for each answer. The answer dataset was only accessible by LD (who created the survey) and the Université Paris Cité Information Technology (IT) department/data protection office (who manages the access to the LimeSurvey system). The characteristics of these women concerning menstruation, age, hormonal contraception use, menstrual cup use, menstrual blood donation, heavy menstrual bleeding, dysmenorrhea, conception, endometriosis, and its subtype are depicted in Sup Table 1. Summary results of this survey will be communicated in French using the same communication channels.

**Table 1:** Characteristics of included participants, with or without endometriosis (declarative)

Characteristics (*n* = 433)	With endometriosis (*n* = 299)	Without endometriosis (*n* = 134)	χ^2^ test
**Age**			***p*** **< 0.001**
< 23	9 (3%)	12 (9%)	
23–27	23 (8%)	32 (24%)	
28–32	50 (17%)	21 (16%)	
33–37	112 (37%)	27 (20%)	
> 37	105 (35%)	42 (31%)	
**Hormonal contraception use**	(*n* = 295)*		*p* = 0.76
Yes	80 (27%)	39 (29%)	
No	215 (73%)	95 (71%)	
**Menstrual cup use**			*p* = 0.14
Yes	58 (19%)	18 (13%)	
No but ready to try it	77 (25%)	45 (34%)	
No and don't want to try	154 (52%)	71 (53%)	
**Menstrual blood donation**			***p*** **< 0.001**
Yes	247 (83%)	91 (68%)	
No	52 (17%)	43 (32%)	
**Heavy menstrual bleeding**	(*n* = 285)*	(*n* = 129)*	***p*** **< 0.001**
Yes	177 (62%)	52 (40%)	
No	108 (38%)	77 (60%)	
**Dysmenorrhea**		(*n* = 133)*	***p*** **< 0.001**
Yes	246 (82%)	31 (23%)	
Occasionally	46 (15%)	68 (51%)	
No	7 (2%)	34 (26%)	
**Trying to conceive**	(*n* = 279)*	(*n* = 133)*	***p*** **< 0.001**
Yes	104 (37%)	10 (8%)	
No	175 (63%)	123 (92%)	
**Endometriosis subtype**	(*n* = 297)*		
SUP	33 (11%)	NA	
OMA	41 (14%)	NA	NA
DIE	191 (64%)	NA	
Unknown	32 (11%)	NA	

*When some question had missing data, the actual number of women who responded is indicated

*SUP* superficial endometriosis, *OMA* ovarian endometriosis (endometrioma), *DIE* deep infiltrating endometriosis

### Survey

The questionnaire contained ten questions with defined choices (2 to 5) concerning age, menstruation, menstrual cup use, menstrual blood donation, heavy menstrual bleeding, menstrual pain, hormonal contraception use, current desire to conceive, endometriosis, and endometriosis subtype. No question was mandatory. Our main goal was to assess if women affected or not by endometriosis would have a similar willingness for menstrual blood donation for biological research on endometriosis. We also wanted to see if some characteristics such as dysmenorrhea or hormonal contraception use would be a barrier for menstrual blood donation.

For endometriosis subtype, as the answer is declarative, we expect that mixed subtype will be categorized as the most severe form (DIE > OMA > SUP) which would reflect what is communicated in practice in France (this way, someone with both SUP and OMA will be categorized as OMA, someone with OMA and DIE will be categorized as DIE).

The ten questions and possible answers of the survey (translated in English) are detailed in Supplementary Table 2.

### Data Analysis and Statistics

**Fig. 1:**
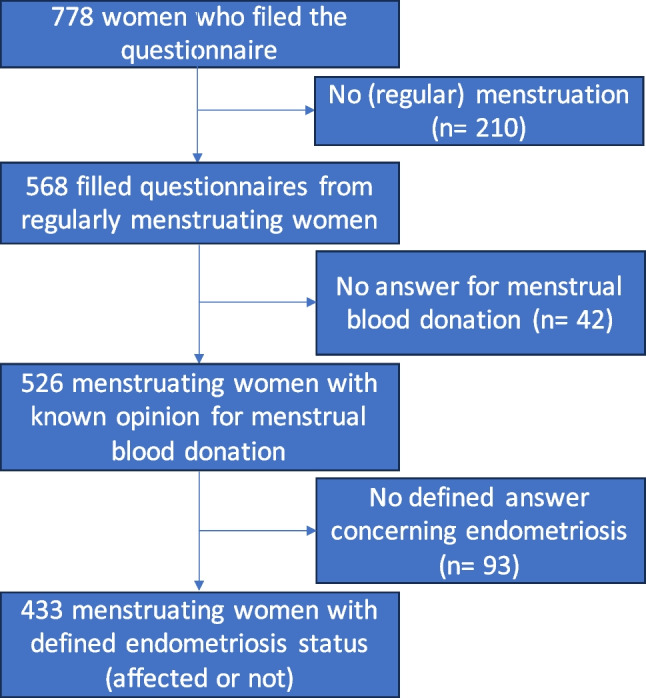
Flowchart to select relevant population

Data were extracted as a csv file, and statistical analysis was performed using R software version R.4.0.3. The primary objective was to evaluate if women with self-declared endometriosis were as likely as unaffected women to be willing to donate menstrual blood. Among the women who answered at least one question of the survey, only the women who had regular menstruation, who expressed their opinion about menstrual blood donation, and had a defined endometriosis status (who declared an established diagnosis or no endometriosis) were included (see flowchart in Fig. 1). The secondary objectives were to assess if some characteristics were predictive of the willingness or unwillingness to donate menstrual blood. Descriptive statistics, such as frequency, were used to present the characteristics of the participants. Pearson’s Chi-squared tests (with Yates’ continuity correction for variables with only two possible answers) were performed to investigate significant associations between endometriosis or willingness to donate menstrual blood and the other factors assessed in the survey. Discrete values were transformed to integers (no as 0, yes as 1, central value for age range, 0.5 for occasional pelvic pain during menstruation, and 0, 1, 2, and 3 for endometriosis subtypes: no endometriosis, superficial, endometrioma, and deeply infiltrating, respectively). A correlation analysis was performed, and for variables strongly correlated with one another (with a correlation coefficient of > 0.6), only one of the variables was kept for downstream analysis (subtype of endometriosis was thus ignored, and only presence or absence of endometriosis was kept). Questionnaires with missing data were eliminated, which resulted in 392 women with a complete set of answers. A generalized linear model was built to assess predictive variables for menstrual blood donation, with a stepwise model selection procedure that iteratively removed the less predictive variable until the model could not be improved anymore.

## Results

### Study Population and Characteristics

During the study period (6 months, with 90% of the answers received during the first month), 778 women filed the online survey. Five hundred sixty-eight women (73%) had regular menstruations (around every month), and 210 (27%) did not. As it was stated in the diffusion material and the short introduction at the top of the survey that the questionnaire was for women with menstruation, it suggests that the 27% of “no” answer correspond to women with irregular menstruation, rather than no menstruation at all. But as it was not further assessed, we focused our analysis on the regularly menstruated women (Fig. 1). Menstrual blood donation for research was generally well accepted with 78% of women ready to donate. However, the menstrual cup is not a very popular item of menstrual hygiene as only 18% of the participants used it (either regularly or occasionally). Regarding to endometriosis, more than half of the women who answered our survey (58%) were affected by the disease.

In the regularly menstruating population, 42 women (7.4%) did not answer at the question for menstrual blood donation, so they were removed from the downstream analyses as it was central to our study. Among the 526 regularly menstruating women who expressed their opinion on menstrual blood donation, 93 women (17.7%) did not have a defined answer concerning endometriosis diagnosis (either no answer, suspected endometriosis, or did not know), and they also were excluded from downstream analyses. A total of 433 women had a defined status for endometriosis (affected with a confirmed diagnosis or not) and gave opinion about menstrual blood donation (Fig. 1, Table 1).

### Endometriosis Patients’ Characteristics

**Fig. 2:**
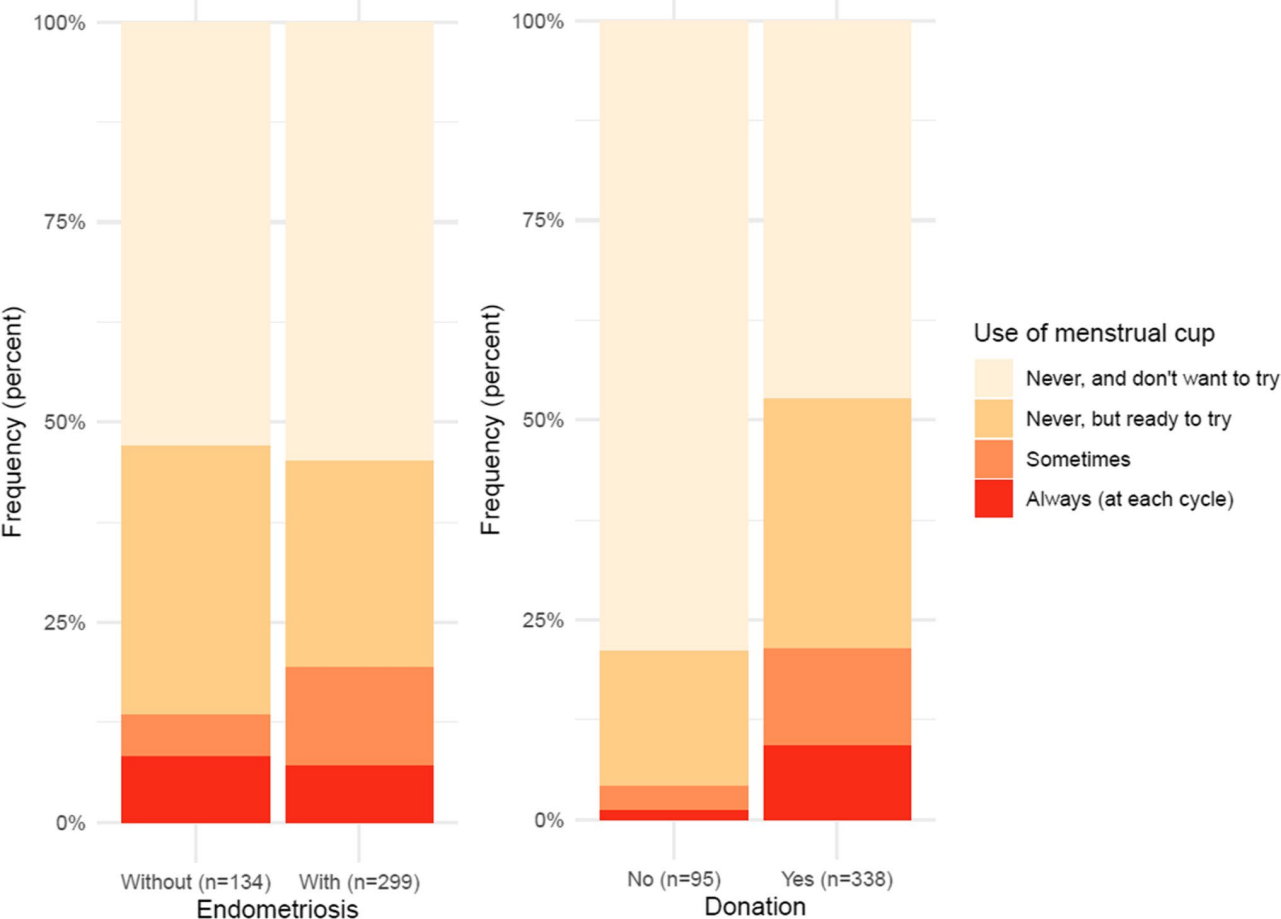
Distribution of women according to their use of a menstrual cup. Left panel: Menstrual cup use and endometriosis status. Right panel: Menstrual cup use and willingness to donate menstrual blood

When comparing women who self-declared with and without endometriosis (Table 1), we could see that there is a significant difference in the age distribution with an overrepresentation of women in the 33–37 age range and fewer younger women below 28 years old in the endometriosis group (*p* < 0.001). There is also a significant association between the endometriosis status and willingness to donate menstrual blood: women with endometriosis are more likely to donate their menstrual blood (83% of women with endometriosis versus 68% of women with endometriosis, *p* < 0.001). Endometriosis patients also exhibited more often heavy menstrual bleeding (62% of endometriosis women versus 40% in unaffected women, *p* < 0.001). Unsurprisingly, women with endometriosis are more likely to experience dysmenorrhea, and in particular, they suffer from pelvic pain at every menstruation (82%) compared to women without endometriosis (23%, *p* < 0.001). Of note, dysmenorrhea was common in unaffected women with half of them (51%) occasionally experiencing pelvic pain during menstruation, in addition to the 23% experiencing it every month. While there was no difference with endometriosis affected and unaffected women concerning hormonal contraception use, endometriosis patients were more often trying to conceive (37% vs 8%, *p* < 0.001). Endometriosis affected and unaffected women were as likely to use a menstrual cup (Fig. 2). However, the majority of women in both populations are not using menstrual cups and are not willing to use it.

### Factors Associated with Willingness to Donate Menstrual Blood Donation

In addition to the endometriosis status as described above, we were able to highlight associations between other variables and menstrual blood donation (Table 2). Indeed, women experiencing dysmenorrhea every month were more likely to be willing to donate menstrual blood (68 vs 51%) than women never experiencing it or only occasionally (*p* < 0.01).

**Table 2:** Characteristics of women willing or not to donate menstrual blood

Menstrual blood donation	Yes (*n* = 338)	No (*n* = 95)	χ^2^ test
**Age**			*p* = 0.115
< 23	17 (5%)	4 (4%)	
23–27	41 (12%)	14 (15%)	
28–32	53 (16%)	18 (19%)	
33–37	119 (35%)	20 (21%)	
> 37	108 (32%)	39 (41%)	
**Hormonal contraception use**	(*n* = 335)*	(*n* = 94)*	*p* = 0.053
Yes	85 (25%)	34 (36%)	
No	250 (75%)	60 (64%)	
**Menstrual cup use**			***p*** **< 0.001**
Yes	72 (21%)	4 (4%)	
No, but willing to try	106 (31%)	16 (17%)	
No and don't want to try	160 (47%)	75 (79%)	
**Heavy menstrual bleeding**	(*n* = 321)*	(*n* = 93)*	*p* = 0.486
Yes	181 (56%)	48 (52%)	
No	140 (44%)	45 (48%)	
**Dysmenorrhea**		(*n* = 94)*	***p*** **< 0.01**
Yes	229 (68%)	48 (51%)	
Occasionally	84 (25%)	30 (32%)	
No	25 (7%)	16 (17%)	
**Trying to conceive**	(*n* = 319)*	(*n* = 93)*	*p* = 0.057
Yes	96 (30%)	18 (19%)	
No	223 (70%)	75 (81%)	
**Endometriosis**			***p*** **< 0.001**
Yes	247 (73%)	52 (55%)	
No	91 (27%)	43 (45%)	
**Endometriosis subtype** ^**a**^	(*n* = 245)*	(*n* = 52)*	*p* = 0,160
SUP	26 (11%)	6 (12%)	
OMA	38 (16%)	3 (6%)	
DIE	157 (64%)	34 (65%)	
Unknown	24 (10%)	9 (17%)	

*When some question had missing data, the actual number of women who responded is indicated

*SUP* superficial endometriosis; *OMA* ovarian endometriosis (endometrioma); *DIE* deep infiltrating endometriosis

^a^For women who declared a confirmed endometriosis diagnosis

A strong limiting factor seems to be related to the use of a menstrual cup. Indeed, women who do not want to try a menstrual cup are less willing to donate their menstrual blood (47 vs 79%) compared to women ready to try it or already using it (*p* < 0.001).

There were also tendencies with fewer women who use hormonal contraception likely to donate menstrual blood (25% vs 36%, *p* = 0.053) and more women that are trying to conceive likely to donate menstrual blood (30% vs 19%, *p* = 0.057).

Age and abundance of the menstrual blood flow did not have an impact on menstrual blood donation. Within women with endometriosis, the subtype of endometriosis did not affect the willingness to donate menstrual blood.

### Correlation Between Factors

**Fig. 3:**
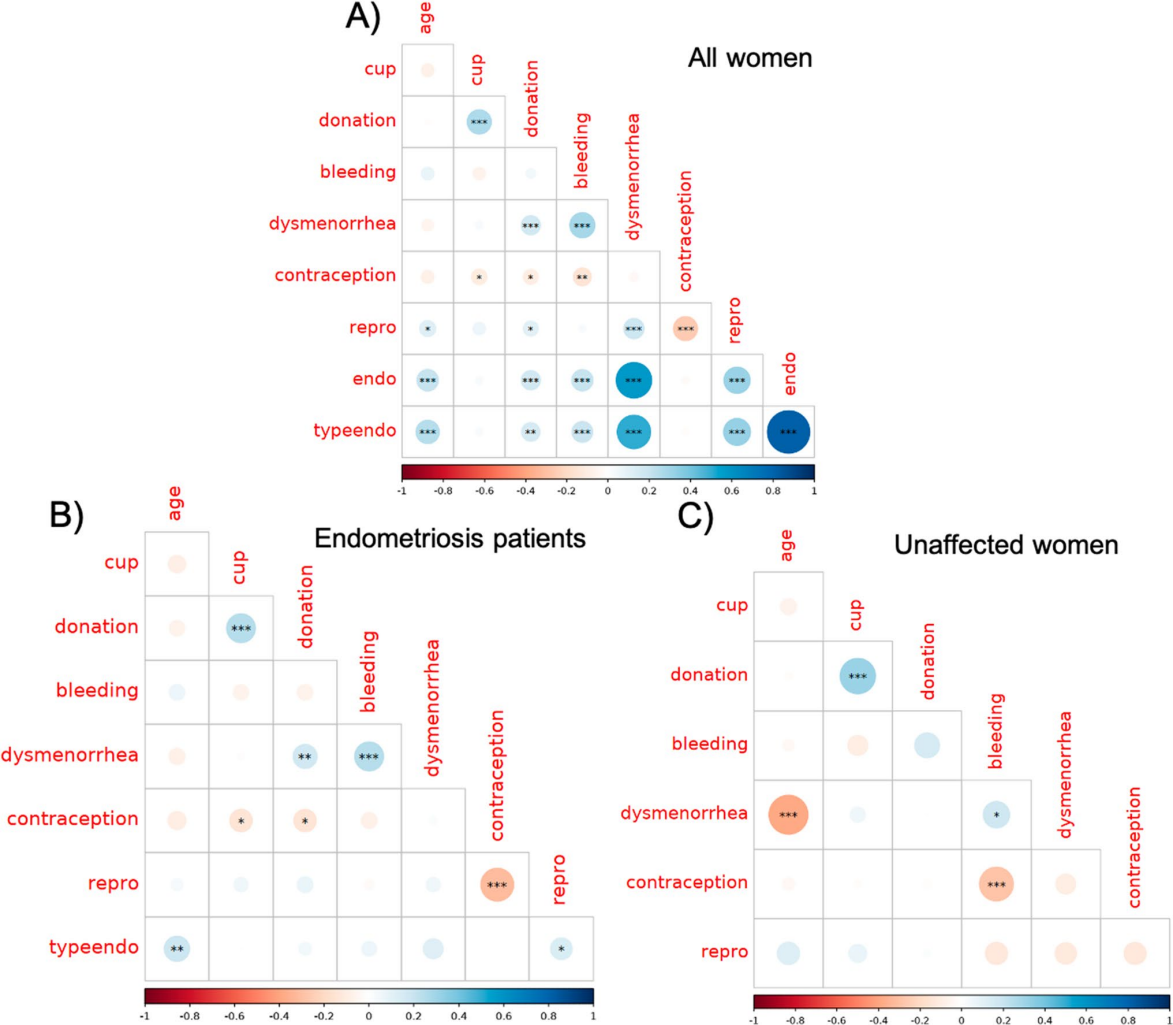
Graphical representation of the correlation matrix. **A** Graphical representation of the correlation matrix for all the studied variables in all women (*n* = 392 to 433, as some data were missing). **B** Graphical representation of the correlation matrix for the indicated variables in women with self-declared endometriosis (*n* = 279 to 299). **C** Graphical representation of the correlation matrix for the indicated variables in self-declared unaffected women (*n* = 129 to 134). The size and color of the dots represent the correlation coefficient value (see color scale). Pearson statistical test and adjusted *p*-value using the FDR (false discovery rate) method. *p < 0.05, **p < 0.01, and ***p < 0.001. Here, the name of the variables was shortened to allow an easier readability: “cup” stands for menstrual cup use, “donation” for willingness to donate menstrual blood, “bleeding” for heavy menstrual flow, “dysmenorrhea” for pelvic pain during menstruations, “contraception” for taking hormonal contraception, “repro” for trying to conceive, and “typeendo” for the type of endometriosis

After transforming variables to numerical value, a correlation matrix was generated, and correlation between all variables is further detailed in Fig. 3. None of the variables, except endometriosis status and endometriosis subtype, displayed a correlation coefficient greater than 0.6, the threshold used to eliminate a variable for the subsequent generalized linear model. In addition to the associations already described above between endometriosis and willingness to donate menstrual blood with the other variables, there is significant correlation between a few other variables (Fig. 3A). Some were expected, such as the negative correlation between hormonal contraception and trial for conception (*r* =  − 0.26, *p* < 0.001), as well as hormonal contraception and heavy menstrual bleeding (*r* =  − 0.17, *p* < 0.01). There was also a positive correlation between age and trial for conception (*r* = 0.13, *p* < 0.05). Heavy menstrual flow and dysmenorrhea are also positively correlated (*r* = 0.29, *p* < 0.001).

Among women with self-declared endometriosis (Fig. 3B), no new correlation could be observed. Of note, the negative correlation between hormonal contraception and heavy menstrual bleeding that was found in the whole population was not observed in the endometriosis subgroup, neither was the positive correlation between the trial for conception and dysmenorrhea. When taking into account the subtype of endometriosis in affected women, only age was still positively correlated (*r* = 0.18, *p* < 0.01), indicating that the other correlations with menstrual blood donation, heavy menstrual bleeding, and dysmenorrhea are associated with the presence of endometriosis but not its subtype.

Among women who self-declared being unaffected by endometriosis (Fig. 3C), there is a specific correlation that could be observed: a significant negative correlation between age and dysmenorrhea (*r* =  − 0.38, *p* < 0.001), with young people more affected by dysmenorrhea.

### Predictive Factors

**Fig. 4:**
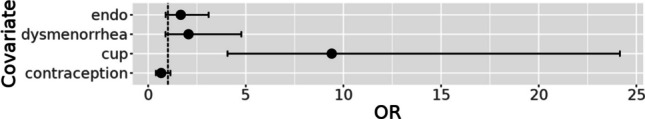
Menstrual cup use predicts willingness to donate menstrual blood. A generalized linear model was built to assess predictive variables for menstrual blood donation from 392 complete set of answers, with a stepwise model selection procedure that iteratively removed the less predictive variable until the model could not be improved anymore. Odds ratio (OR) for each retained covariates and confidence intervals (2.5–97.5%) are plotted. Here, the name of the variables was shortened to allow an easier readability: “endo” stands for endometriosis diagnosis, “dysmenorrhea” for pelvic pain during menstruations, “cup” stands for menstrual cup use, and “contraception” for the use of a hormonal contraception

We used generalized linear model with a stepwise selection procedure to identify potential predictive factors of menstrual blood donation (Fig. 4). The only factor that is significantly predictive in our study is the use of menstrual cup (*p* < 0.001): women using a menstrual cup are indeed nine times more likely to be willing to donate menstrual blood (95% confidence interval [4.065:24.155], Fig. 4). Experiencing dysmenorrhea and an endometriosis diagnosis have a tendency to predict the willingness to donate menstrual blood (95% confidence interval [0.890:4.774] and [0.891:3.094], respectively).

## Discussion

In this study, we showed that women with self-declared endometriosis were more likely to donate their menstrual blood for endometriosis research, than self-declared unaffected women. While the implication towards endometriosis research is without doubt a strong motivation, it is reassuring as women with endometriosis face many challenges [24, 25], and this could negatively influence their motivation.

Concerning menstrual blood donation, we were able to see that the use of a menstrual cup or readiness to use it was a predictive factor. As endometriosis does not seem to influence the use of a cup, nor the willingness to wear one, this is also reassuring. This was not expected as one could think endometriosis women, who often suffer from dyspareunia, would be less likely to wear a menstrual cup that need to be inserted in the vagina. However, as dyspareunia was not evaluated in our questionnaire, we cannot rule out that a severe one would influence the willingness to wear a cup and/or donate menstrual blood. Reassuringly, we did not see any correlation between the use of a cup and the type of endometriosis, which may be considered a proxy for more severe dyspareunia as this symptom correlates with the disease severity [26]. Of note, within the women who never used a menstrual cup, a majority do not want to try it, indicating that while donating menstrual blood for research is largely accepted, a large portion of women will not carry a menstrual cup to do it. This could be linked to an uncomfortable feeling when seeing/manipulating menstrual blood, in addition to the actual use of the cup.

We showed that the women who self-declared as affected by endometriosis were older than unaffected women. This could be a reflection of the long diagnosis delay [5]. In these patients, there was also a significant positive correlation between age and the severity of the disease, which suggest that the disease progress with age, which is a debated subject [27–29]. Endometriosis patients also declared having more heavy menstrual bleeding than unaffected women, which is consistent with previously described results [30, 31].

In women who self-declared as unaffected, we observed an inverse correlation between dysmenorrhea and age, showing higher prevalence of menstrual pain in younger adults, which may suggest undiagnosed young women and is concerning. And while we let the option to answer “suspected endometriosis” in the questionnaire, it was rarely used and quite homogeneously across the different age range. The pain intensity was not assessed, so we cannot know if this influences the suspicion of endometriosis in young adults.

In our population, we observed more women with self-declared endometriosis that are trying to conceive, compared to unaffected women, while the use of hormonal contraception was similar in both groups. This indicates that women with endometriosis often do not use hormonal contraception for this specific reason, which is expected as hormonal medication is the first line of treatment for endometriosis [32]. It may also reflect their difficulties to have and maintain pregnancies [4, 33], so they may be in the “trying to conceive a child” period for longer.

Our data have been obtained from women willing to complete the survey, which may introduce a selection bias, such as women more comfortable talking about the menstruation. Questionnaires were filled anonymously, which is considered to provide more honest answers [34], but prevented us to check that each answer came from a unique person. Questionnaire was filled online, which creates a bias for a population who has an easy access to Internet and so a socioeconomic bias. In France, 92.5% of the households have an access to Internet in 2022 according to the national institute of statistics and economical studies. Another limitation is the sampling of endometriosis affected women, who were certainly reached in majority through the diffusion of the survey by a patient association. It was a great way to connect with affected women, but as member of this association, they may represent a specific subset of endometriosis patient that are especially involved in increasing the knowledge about endometriosis. While it is important to recognize these limitations, there is no reason to believe the sample is nonrepresentative of the endometriosis patients and of the female French population.

In conclusion, we show that, in France, a large majority of women are willing to donate menstrual blood for biomedical research on endometriosis. Reassuringly, endometriosis patients are willing to donate menstrual blood, and dysmenorrhea was not identified as a limiting factor for such donation. The main barrier we could identify is the refusal to use a menstrual cup.

### Supplementary Information

Below is the link to the electronic supplementary material.

Supplementary file1 (DOCX 15 KB)

Supplementary file2 (DOCX 15 KB)

## Data Availability

Raw data are submitted as a supplementary csv file.

## Code Availability

NA (R script available upon request).
