# Supplementary material for: Menstrual Blood Donation for Endometriosis Research: A Cross-Sectional Survey on Women’s Willingness and Potential Barriers
Source: Reprod Sci. 2024 Feb 28;31(6):1617–25. doi: 10.1007/s43032-024-01481-3 (PMC11111534; doi:10.1007/s43032-024-01481-3)
Supplement: Supplementary file 1 — Supplementary file1 (DOCX 15 KB) [file 43032_2024_1481_MOESM1_ESM.docx]

| **Supplementary Table 1**: Characteristics of all participants | |  |  |  |
| --- | --- | --- | --- | --- |
| Characteristics | All (n=778, 100%) |  |  |  |
| Regular menstruation (n=778) | |  |  |  |
| Yes | 568 (73%) |  |  |  |
| No | 210 (27%) |  |  |  |
| Age (n=778) | |  |  |  |
| <23 | 41 (5%) |  |  |  |
| 23-27 | 122 (16%) |  |  |  |
| 28-32 | 126 (16%) |  |  |  |
| 33-37 | 213 (27%) |  |  |  |
| >37 | 276 (35%) |  |  |  |
| Hormonal contraception use (n=650) |  |  |  |  |
| Yes | 259 (40%) |  |  |  |
| No | 391 (60%) |  |  |  |
| Menstrual cup use (a) (n=526) | |  |  |  |
| Yes | 94 (18%) |  |  |  |
| No but ready to try it | 157 (30%) |  |  |  |
| No and don't want to try | 275 (52%) |  |  |  |
| Menstrual blood donation (a) (n=526) | |  |  |  |
| Yes | 409 (78%) |  |  |  |
| No | 117 (22%) |  |  |  |
| Heavy menstrual bleeding (a) (n=501) | |  |  |  |
| Yes | 287 (57%) |  |  |  |
| No | 214 (43%) |  |  |  |
| Dysmenorrhea (a) (n=524) | |  |  |  |
| Yes | 337 (64%) |  |  |  |
| Occasionally | 141 (27%) |  |  |  |
| No | 46 (9%) |  |  |  |
| Trying to conceive (a) (n=443) | |  |  |  |
| Yes | 119 (24%) |  |  |  |
| No | 378 (75%) |  |  |  |
| Endometriosis (a) (n=515) | |  |  |  |
| Yes (confirmed diagnosis) | 299 (58%) |  |  |  |
| Suspected endometriosis | 27 (5%) |  |  |  |
| Don't know | 55 (11%) |  |  |  |
| No | 134 (26%) |  |  |  |
| Endometriosis subtype (a, b) (n=297) | |  |  |  |
| SUP | 32 (11%) |  |  |  |
| OMA | 41 (14%) |  |  |  |
| DIE | 191 (64%) |  |  |  |
| Unknown | 33 (11%) |  |  |  |
| (a) only women with regular menstruation were taken into account | | | | |
| (b) only women with a confirmed endometriosis diagnosis were taken into account | | | | |
