# Supplementary material for: Menstrual Blood Donation for Endometriosis Research: A Cross-Sectional Survey on Women’s Willingness and Potential Barriers
Source: Reprod Sci. 2024 Feb 28;31(6):1617–25. doi: 10.1007/s43032-024-01481-3 (PMC11111534; doi:10.1007/s43032-024-01481-3)
Supplement: Supplementary file 2 — Supplementary file2 (DOCX 15 KB) [file 43032_2024_1481_MOESM2_ESM.docx]

**Supplementary Table 2: detailed survey questions and possible answers**

| Questions | Possible answers | | | | |
| --- | --- | --- | --- | --- | --- |
| 1)    How old are you? | 22 or less | 23-27 years old | 28-32 years old | 33-27 years old | 38 or more |
| 2)    Do you have menstruations every month (roughly)? | Yes | No |  | | |
| 3)    During your menstruation, do you use a menstrual cup? | Always* (at least one day of each menstruation) | Occasionally* | Never, but I would be ready to try it | Never, and I don’t want to try it |  |
| 4)    Would you donate your menstrual blood for a research project? | Yes | No |  | | |
| 5)    Do you have a heavy menstrual flow? | Yes | No |  | | |
| 6)    Are you experiencing pelvic pain during your menstruation (with a need to use antalgic medication)? | Yes (every month) | Sometimes | No |  | |
| 7)    Do you use a hormonal contraception? | Yes | No |  | | |
| 8)    Are you trying to get pregnant? | Yes | No |  | | |
| 9)    Do you have endometriosis? | Yes (established diagnosis) | Suspected endometriosis | No | I do not know |  |
| 10) If yes, do you know which type? | Superficial | Ovarian (endometrioma) | Deeply infiltrating | I do not know |  |
| *For the use of the menstrual cup, the “Always” and “Occasionally” answers were regrouped as “Yes” to avoid very small instances in these groups. | | | | | |
